# Disruption of Memory Reconsolidation Erases a Fear Memory Trace in the Human Amygdala: An 18-Month Follow-Up

**DOI:** 10.1371/journal.pone.0129393

**Published:** 2015-07-01

**Authors:** Johannes Björkstrand, Thomas Agren, Andreas Frick, Jonas Engman, Elna-Marie Larsson, Tomas Furmark, Mats Fredrikson

**Affiliations:** 1 Department of Psychology, Uppsala University, Uppsala, Sweden; 2 Department of Radiology, Department of Surgical Sciences / Radiology, Uppsala University, Uppsala, Sweden; 3 Department of Clinical Neuroscience, Karolinska Institutet, Stockholm, Sweden; University of Lethbridge, CANADA

## Abstract

Fear memories can be attenuated by reactivation followed by disrupted reconsolidation. Using functional magnetic resonance imaging we recently showed that reactivation and reconsolidation of a conditioned fear memory trace in the basolateral amygdala predicts subsequent fear expression over two days, while reactivation followed by disrupted reconsolidation abolishes the memory trace and suppresses fear. In this follow-up study we demonstrate that the behavioral effect persists over 18 months reflected in superior reacquisition after undisrupted, as compared to disrupted reconsolidation, and that neural activity in the basolateral amygdala representing the initial fear memory predicts return of fear. We conclude that disrupting reconsolidation have long lasting behavioral effects and may permanently erase the fear component of an amygdala-dependent memory.

## Introduction

Etiological processes in the anxiety disorders involve amygdala-dependent memory mechanisms that link stressful events to previously neutral stimuli [1] by means of fear conditioning [2–3]. Consequently, amygdala hypersensitivity characterize the anxiety disorders [4–5], which are highly prevalent [6], cause great suffering [7] and high societal costs [8]. Pharmacological and behavioral treatments, even though they reduce amygdala activity [9], have limited long-term success because relapses occur [10–11]. When fear is extinguished it can be renewed by contextual alterations, re-occur spontaneously, and is easily re-acquired [12–13]. It has been argued that fear memories can be effectively updated as safe by preventing their reconsolidation through extinction training within a critical reconsolidation time window [13–14]. Disrupting reconsolidation attenuates or even removes the fear component of the memory, and this effect persists after one year [14]. While the short-term effects of disrupted reconsolidation seem amygdala dependent both in rodents and humans [13–17], the long-term effects have not been linked to amygdala activity. Here we test if amygdala activity reflecting reconsolidation is predictive of return of fear over 18 months and if the behavioral effect is stable over time.

Experimental groups previously given extinction inside or outside of the reconsolidation interval [16] respectively were compared for return of fear 18 months after extinction using a behavioral reacquisition protocol. This measure was chosen because previous studies both in animals [13] and humans [18] have demonstrated that reacquisition is compromised in groups with disrupted reconsolidation but intact when memories are reconsolidated after activation. Also, in anxiety disorders reacquisition frequently occur and has great clinical relevance.

We first evaluated if the behavioral effect of memory reconsolidation persists over 18 months. Then, we related fear memory savings (e.g. [19]) to initial amygdala activity to determine if the long-term effects of undisrupted reconsolidation can be predicted from activity in areas of the basolateral amygdala representing the fear memory trace, while leaving disrupted reconsolidation uncorrelated to fear memory trace activity [16]. Based on Schiller et al. [14], we predicted that return of fear would occur in the group with undisrupted reconsolidation but not in the disrupted group and, based on Agren et al. [16], that regions in the amygdala representing the initial fear memory trace would predict the strength of return of fear.

## Methods

### 2.1 Participants, stimuli, materials and procedure

Originally, we recruited thirty participants who completed a discriminative fear-conditioning session consisting of 16 pairings of a conditioned stimulus (CS+) with an electrical shock unconditioned stimulus (UCS) and 16 unpaired presentations of CS- unrelated to the UCS. The CS were presented for 6s with UCS delivery at 250 ms prior to CS+ termination with an intertrial interval of 14s. The conditioned stimulus predicting the UCS consisted of a picture of a lamp lighting up in blue or red, contrabalanced across subjects (see Agren et al. [16] for details). Those who acquired fear conditioning (n = 22) continued the experiment, while those who did not were dismissed [16]. The next day subjects were randomly assigned either to a 10min or a 6h group. In the 10min group, extinction training consisting of 8 CS+ and 8 CS- presentations was performed 10 minutes after a 2 minute fear memory reminder, hence inside of the reconsolidation interval. The 6h group performed extinction in the same vein, but 6 hours after the memory reminder, i.e. outside of the reconsolidation interval. No differences in fear acquisition or extinction were found between the two groups [16]. On day 3, a renewal session was conducted in the fMRI scanner consisting of 8 presentations of each CS cue and two days later, a reinstatement session included un-signaled shock presentations and re-exposure to conditioning cues. The electric shocks were administered by the PsychLab SHK 1 shock stimulator (PsychLab, Cambridge, MA) and had a maximum strength of 5mA. Electrodes (EL124), prepared with salt free CEFAR electrolyte medium to facilitate shock conduction, were attached with surgical tape to the right dorsal lower arm of the participants.

### 2.2 Re-test procedure

**Fig 1:**
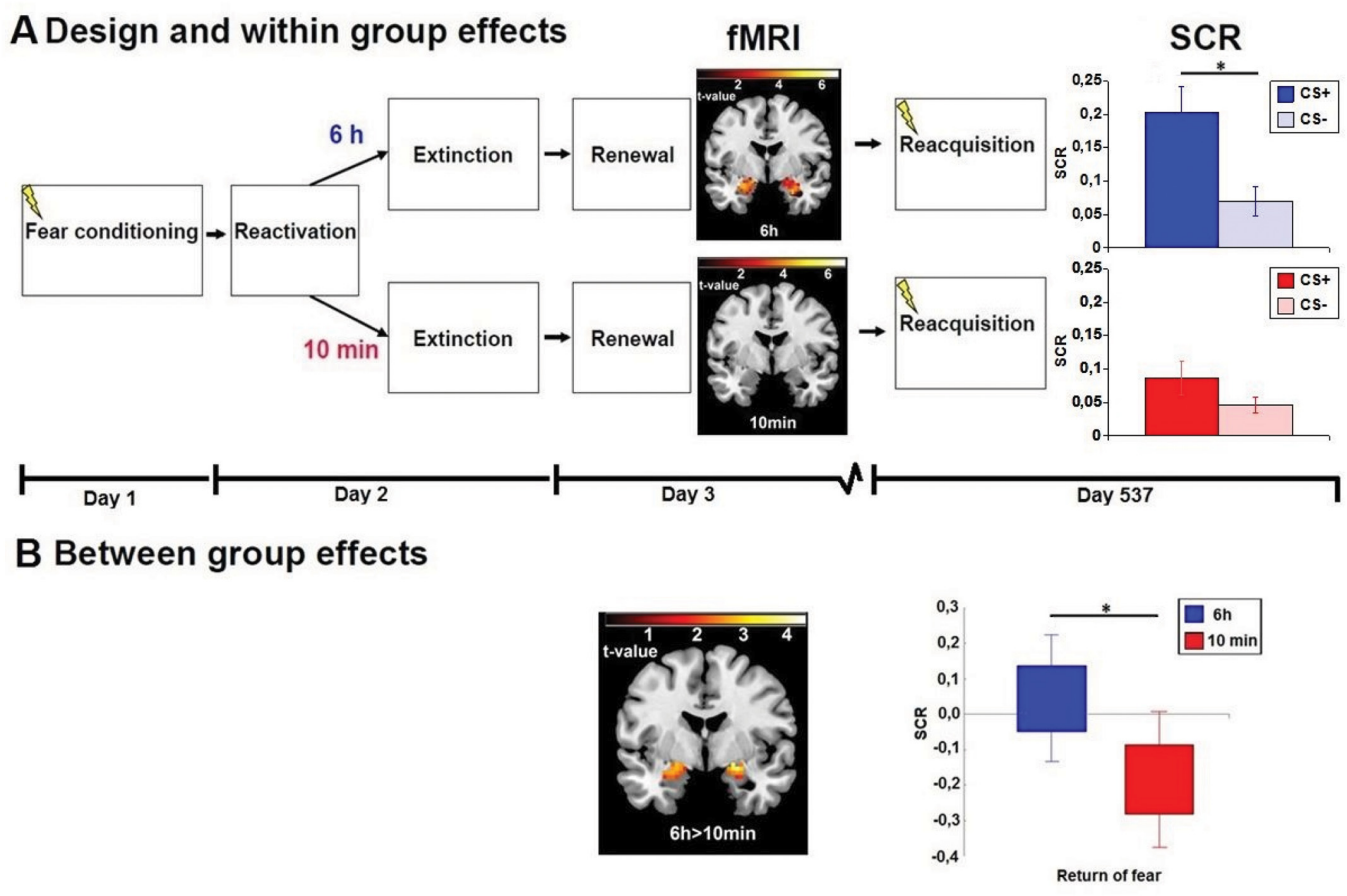
Amygdala activity predicts return of fear over 18 months. A) Fear conditioning on day 1 was established by pairing a visual cue with electric shocks and then the memory reminder was given on day 2 either 10 min or 6 hours prior to extinction was performed, through exposure to the conditioned cue without shocks. On day 3, memory related amygdala activity was evaluated using functional magnetic resonance imaging (fMRI) during renewal-induced fear, and return of fear was evoked on day 537. Skin conductane respones (SCR, the electrophysiological fear index) in the 6h group with undisrupted reconsolidation, but not the 10min group with disrupted reconsolidation, discriminated between the shock-reinforced (CS+) and unreinforced (CS-) cue during reacquisition. See the two right hand bars in row A. Bars represent means and error-bars are SEM. Return of fear was predicted by initial neural activity in the basolateral amygdala in the 6h but not the 10min group. (Coronal brain slices in the two top rows). B) As illustrated in the right panel in row B, return of fear was stronger after undisrupted (6h) than disrupted (10min) reconsolidation at 18 months follow-up, as reflected in enhanced reactivity to the cue predicting shocks. Boxes illustrate mean± SEM, whiskers represent SEMx1.96. The coupling between the electrophysiological fear measure and brain activity was significantly stronger in the 6h than in the 10min group as reflected by enhanced connectivity between SCR and BOLD activity in the basolateral amygdala; mapped in the coronal brain slice in row B. * indicates p<.05 one-tailed. The right side of the brain is depicted to the right.

We re-tested, 20 of the original 22 participants after 18 months (mean = 537 days, SEM = 7.6 days) but had to exclude one participant in the 10min group due to equipment failure. See Fig 1A for design and time-lines. To evaluate return of fear the original CS+ was again paired with 4 UCS presentations during reacquisition while skin conductance responses (SCRs) were recorded. Identical both to Schiller and co-workers [14] and to Agren et al. [16] return of fear was evaluated as the increase in SCRs from the last extinction trial to responses obtained during reacquisition. The return of fear-score was calculated by subtracting the last SCR during extinction at day 2 from the average SCRs to CS+ during reacquisition at day 537. The regional ethical review board at Uppsala University approved the study, and written informed consent was obtained from all participants.

### 2.3 Electrodermal activity

SCRs were measured in μSiemens with the PsychLab 24 bit Skin Conductance System using two 8 mm Ag/AgCl electrodes filled with isotonic electrolyte gel attached to the hypothenar eminence of the left hand [20]. Stimulus induced SCRs were measured by calculating the maximum of the skin conductance deflection in the 1.5–5.75 s interval after stimulus onset subtracted by the immediate preceding baseline of 300ms [20] because this measure has high internal consistency and good temporal stability [21]. To minimize individual variability and maximize the impact of the experimental manipulations, all SCRs were range-corrected by dividing each reaction for every individual with that individual’s maximum deflection, irrespective of stimuli and experimental phase [22]. In contrast, uncorrected scores were used in the analyses evaluating individual differences when performance measures were correlated with brain activity, to optimize between subjects variability. Because of theoretically predicted effects we evaluated results using directed t-tests.

### 2.4 Brain imaging

Brain imaging was performed with a 3T whole body scanner (Philips Achieva 3.0T TX, Philips Medical Systems, Best, The Netherlands) using an 8-channel head coil. Head movement was restricted using foam cushions. Initial scanning was performed to create an anatomical T_1_-weighted reference data set with a voxel size of 0.8×1.0×2.0 mm and 60 slices. During visual presentations blood oxygen level dependent (BOLD) imaging was performed using a single shot EPI sequence with parameters TE/TR 35/3000 ms, flip angle 90°, acquisition matrix 76×77, acquired voxel size 3.0×3.0×3.0 mm. A total of 30 slices were acquired for whole brain coverage. BOLD data were motion corrected, temporally and spatially smoothed using a 6 mm FWHM kernel. Brain coordinates are according to the Montreal Neurological Institute (MNI). The contrast used in BOLD analysis is the first presentation of the CS+ over an immediately preceding baseline. Like in the previous study [16], when correlating BOLD with SCR we used a voxel extension criterion of five consecutive voxels (135 mm^3^) and a statistical threshold of p<.05 uncorrected for multiple comparison. Regions of interest (ROIs) consisted of the bilateral amygdala, defined according to the Automated Anatomical Labelling atlas within the Wake Forest University Pickatlas [23] and for anatomical referencing we used the Mai, Assheur and Paxinos medial temporal lobe atlas [24] with MNI coordinates transformed into Talairach space. To evaluate possible co-localizations between the previous and the present amygdala predictions of fear return, we created a mask using the overlap between the image depicting voxels separating the 6h from the 10min group during renewal on day 3, reflecting the fear memory trace, and the image depicting the correlation between amygdala BOLD during renewal and SCR during the return of fear 5 days after acquisition, i.e. short term return of fear [16]. We then used this mask when analyzing the correlation between amygdala BOLD during renewal and SCR during reacquisition on day 537. Thus, we studied if areas predicting short-term and long term return of fear overlapped with each other and the original memory trace.

## Results

During reacquisition the 6h group discriminated CS+ from CS- [t(9) = 2.08; p = 0.03, one-tailed], while the 10min group did not [t(8) = 0.95; p<1; n.s.]—see Fig 1A right hand panels. Thus, reacquisition occurred in the 6h but not the 10min group. Return of fear was evident in the 6h group characterized by enhanced responding to the CS+ following CS-UCS pairings as compared to the 10min group [t(17) = 1.72; p = 0.05, one-tailed]—see Fig 1B right hand panel. Hence, after 18 months return of fear occurred only in the group with undisrupted reconsolidation.

**Fig 2:**
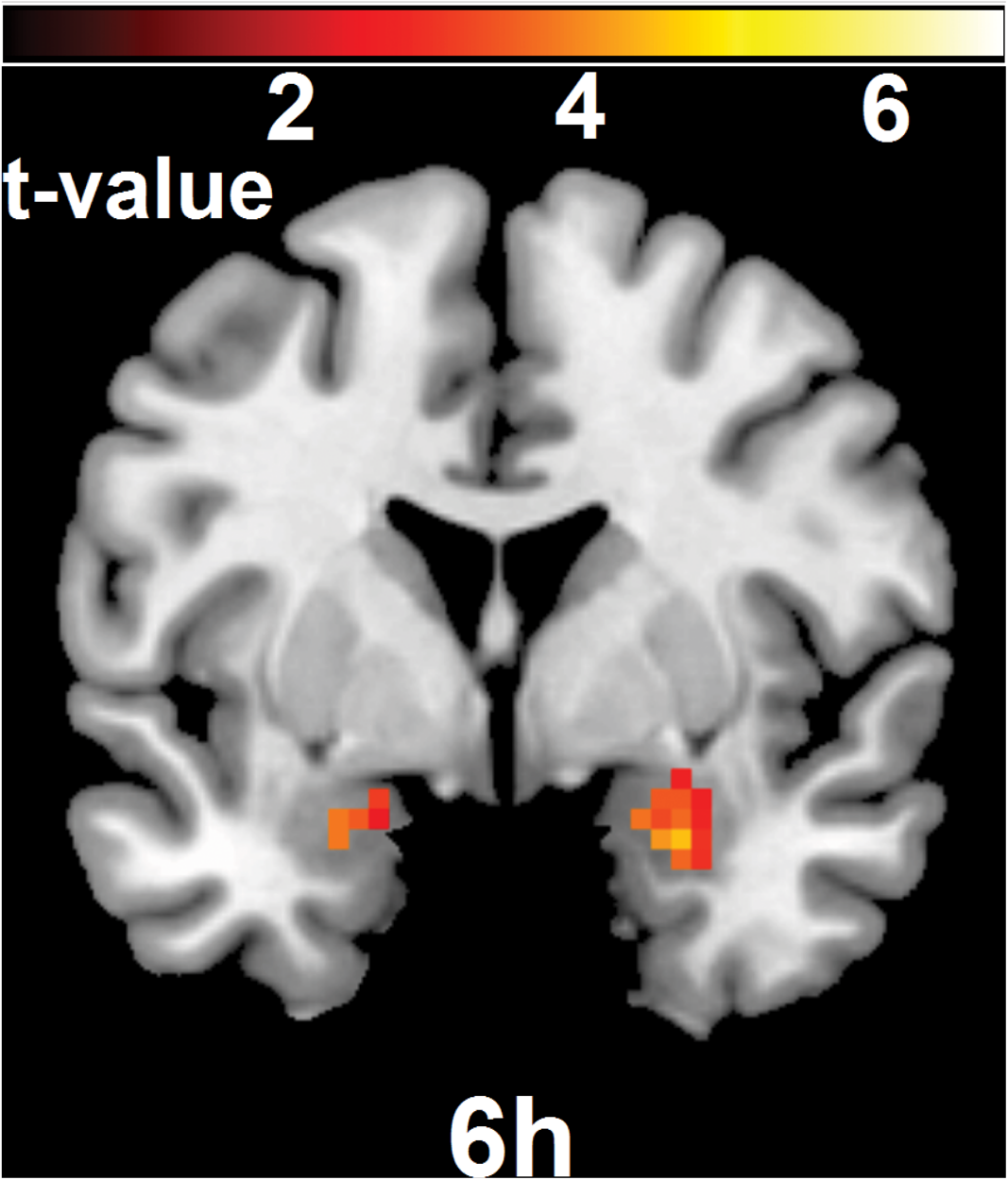
Areas predicting long-term return of fear overlap both with the original fear memory trace and with areas predicting short-term return of fear. Areas in the amygdala in the 6h group representing the original fear memory trace and that overlapped with areas predicting short-term return of fear in turn overlapped with areas predicting long-term return of fear. The right side of the brain is depicted to the right.

Following the same analytic strategy as Agren et al. [16] we next evaluated if initial amygdala activity during short-term renewal, representing the original fear memory trace, predicted long-term fear expression by correlating BOLD activity in the amygdala with return of fear after 18 months. In the 6h group, bilateral amygdala activity predicted return of fear – see Fig 1A upper brain slice (xyz = 30, -4, -20; Z = 3.46; P<0.001; 1620mm^3^; xyz = -30, -1, -17; Z = 3.81; P<0.001; 1539mm^3^), while in the 10min group no correlations between brain activity and electrophysiological fear measures emerged – see Fig 1A lower brain slice. The effects were significant bilaterally also when corrected for multiple comparisons (xyz = 30, -4, -20; Z = 3.46; P_FWE_ = 0.029; 54 mm^3^; xyz = -30, -1, -17; Z = 3.81; P_FWE_ = 0.010; 81 mm^3^). The correlation in the 6h group was significantly stronger than in the 10min group bilaterally in the amygdala (xyz = 24, 2, -14; Z = 3.46; P<0.001; 972mm^3^; xyz = -24, -4, -20; Z = 2.53; P = 0.006; 1242mm^3^). See the brain slice in Fig 1B. The effect survived correction for multiple comparisons in the right amygdala (xyz = 24, 2, -14; Z = 3.46; P_FWE_ = 0.022; 81 mm^3^). To evaluate if these findings were spatially similar to previous results we analyzed if fear predicting areas after 18 months were co-localized with the areas representing the original fear memory trace as well as areas predicting short-term return of fear. Results revealed that areas in the amygdala that predicted return of fear over 18 months in the 6h group were co-localized both with areas in the amygdala reflecting the original memory trace and areas predicting short term return of fear over 5 days (xyz = -24, -4, -20; Z = 3.78; p<0.001; 378mm^3^; xyz = 27, -1, -23; Z = 3.12; p = 0.001; 675mm^3^)—see Fig 2.

## Discussion

Return of fear in the group with undisrupted reconsolidation was predicted by amygdala activity reflecting the localization of the memory trace formed 18 months earlier, while disrupted reconsolidation did not result in return of fear and was unrelated to initial amygdala activation.

This study adds to the human literature on reconsolidation disruption using behavioral means. Since the initial Monfils study [13] demonstrated the feasibility of using extinction training time-locked to a reconsolidation interval in rodents, several independent studies [14], [16–18], [25–26] have conceptually replicated this finding in humans (but see also [27–29]). Schiller et al. [14] demonstrated that extinction training performed within the reconsolidation interval, after a fear memory reminder, led to inhibition of fear. In contrast, extinction training performed outside of the reconsolidation interval spared the memory and fear returned [14]. The effect was present a year later, suggesting that the fear memory was updated as being safe or even erased. In animals, lesion and stimulation studies using pharmacological and physiological probes indicate that the neural functions enabling fear memory formation are located in the lateral amygdala [1], [30–31], while both the lateral and the basal subdivisions have been implicated in reconsolidation disruption [13], [32]. In humans, lesion [33–34] and brain imaging studies [35–40] confirm that the amygdala is a key area for fear memory encoding, and we recently provided support for the hypothesis that fear memory reconsolidation in humans is mediated by the basolateral amygdala[16]. Schiller et al. [17] demonstrated that amygdala reductions followed extinction both inside and outside the reconsolidation interval, and that disrupted extinction did not engage the ventromedial prefrontal cortex (vmPFC), whereas standard extinction performed outside the critical interval depended on prefrontal inhibition. Results from Agren et al. [16] and Schiller et al. [17] are internally consistent in that greater amygdala reductions resulted from extinction inside as compared to outside the reconsolidation interval and in that the vmPFC amygdala coupling was absent in groups given extinction within the reconsolidation interval.

Here, we demonstrate that extinction performed within, but not outside the reconsolidation interval have long lasting effects on fear return reflecting amygdala-dependent fear memory processes. In contrast, extinction training performed outside the reconsolidation widow spared the memory trace and resulted in fear expression when the fear memory was activated 18 months later, supporting enduring long-term memory effects of both disrupted and undisrupted reconsolidation. The present study is, of course, limited by the initial sample size and further limited by loss of 3 participants to follow up. But the a priori hypotheses were confirmed supporting the robustness of the initial effects [16].

## Conclusion

We conclude that the effects of disrupting reconsolidation behaviorally persist up to 18 months. This supports that behavioral disruption of reconsolidation has long lasting effects and may permanently update a fear memory trace as safe [14], [16–18]. The experimental results may open new opportunities for exposure based therapies to prevent relapse over prolonged periods of time or even permanently.
